# IL-4Rα Signaling in Keratinocytes and Early IL-4 Production Are Dispensable for Generating a Curative T Helper 1 Response in *Leishmania major*-Infected C57BL/6 Mice

**DOI:** 10.3389/fimmu.2017.01265

**Published:** 2017-10-10

**Authors:** Marc Descatoire, Benjamin P. Hurrell, Melissa Govender, Katiuska Passelli, Berenice Martinez-Salazar, Ramona Hurdayal, Frank Brombacher, Reto Guler, Fabienne Tacchini-Cottier

**Affiliations:** ^1^Department of Biochemistry, WHO-Immunology Research and Training Center, University of Lausanne, Lausanne, Switzerland; ^2^Division of Immunology and South African Medical Research Council (SAMRC) Immunology of Infectious Diseases, Faculty of Health Sciences, Institute of Infectious Diseases and Molecular Medicine (IDM), University of Cape Town, Cape Town, South Africa; ^3^International Centre for Genetic Engineering and Biotechnology (ICGEB), Cape Town Component, Cape Town, South Africa; ^4^Department of Molecular and Cell Biology, Faculty of Science, University of Cape Town, Cape Town, South Africa

**Keywords:** *Leishmania*, IL-4, IL-4Rα, T helper cell differentiation, T helper 1, T helper 2, keratinocytes, mast cells

## Abstract

Experimental infection with the protozoan parasite *Leishmania major* has been extensively used to understand the mechanisms involved in T helper cell differentiation. Following infection, C57BL/6 mice develop a small self-healing cutaneous lesion and they are able to control parasite burden, a process linked to the development of T helper (Th) 1 cells. The local presence of IL-12 has been reported to be critical in driving Th1 cell differentiation. In addition, the early secretion of IL-4 was reported to potentially contribute to Th1 cell differentiation. Following infection with *L. major*, early keratinocyte-derived IL-4 was suggested to contribute to Th1 cell differentiation. To investigate a putative autocrine role of IL-4 signaling on keratinocytes at the site of infection, we generated C57BL/6 mice deficient for IL-4Rα expression selectively in keratinocytes. Upon infection with *L. major*, these mice could control their inflammatory lesion and parasite load correlating with the development of Th1 effector cells. These data demonstrate that IL-4 signaling on keratinocytes does not contribute to Th1 cell differentiation. To further investigate the source of IL-4 in the skin during the first days after *L. major* infection, we used C57BL/6 IL-4 reporter mice allowing the visualization of IL-4 mRNA expression and protein production. These mice were infected with *L. major*. During the first 3 days after infection, skin IL-4 mRNA expression was observed selectively in mast cells. However, no IL-4 protein production was detectable locally. In addition, early IL-4 blockade locally had no impact on subsequent Th1 cell differentiation and control of the disease. Taken together, the present data rule out a major role for skin IL-4 and keratinocyte IL-4Rα signaling in the development of a Th1 protective immune response following experimental infection with *L. major*.

## Introduction

Upon infection, *Leishmania* protozoan parasites can cause a spectrum of diseases ranging from cutaneous, muco-cutaneous to visceral forms. Following *Leishmania major* experimental infection, C57BL/6 mice develop a small cutaneous lesion that is self-healing. Healing of lesion and control of parasite load were shown to result from the differentiation of CD4^+^ T helper (Th) 1 cells characterized by their secretion of high levels of IFNγ, a cytokine promoting the differentiation of M1 macrophages that kill intracellular parasites. In contrast, following *L. major* infection, BALB/c mice develop non-healing lesions and are not able to control their parasite load. This phenotype was shown to correlate with the development of CD4^+^ Th2 cells secreting IL-4 and IL-13 cytokines (1, 2). These cytokines induce the differentiation of M2 macrophages that favor parasite survival within macrophages (3). The *L. major* experimental model was the first murine model demonstrating that the discovery of Th1 and Th2 cells subsets by Mosmann et al. *in vitro* (4) had some relevance *in vivo*.

It is well established that the IL-12 produced by antigen presenting cells (APC) is necessary to launch the differentiation of CD4^+^ Th1 cells that protect against intracellular pathogens, including *Leishmania* (5). In contrast the role of IL-4 in *L. major* susceptibility and Th2 cell differentiation is more controversial. Following infection with *L. major* (LV39), IL-4^−/−^ or IL-4Rα^−/−^ mice on a BALB/c genetic background were able to control lesion size and the levels of IFNγ present in draining lymph node (dLN) cells was either very low or remained unchanged compared to that observed in BALB/c wild-type mice (6, 7). These data suggested that IL-4 was critical for *L. major* susceptibility and Th2 cell differentiation. The C57Bl/6x129 IL-4^−/−^ mice used in these studies were backcrossed for six generations onto the BALB/c genetic background. In contrast, following infection with *L. major* LV39 IL-4^−/−^ mice generated with embryonic stem cells of BALB/c origin still developed progressive non-healing lesions that were comparable to those of similarly infected wild-type BALB/c mice (8). Infection of these mice with another strain of *L. major* (IR173) resulted in partial control of lesion size in IL-4^−/−^ mice, while IL-4Rα^−/−^ controlled lesion size efficiently (9). Additional studies using IL-4 or IL-4Rα-deficient mice showed that following infection with *L. major* Th2 differentiation could develop in absence of IL-4 (10–12). Specific deletion of IL-4Rα signaling on T cells resulted in a healing phenotype in BALB/c mice associated with increased IFNγ response, suggesting a role for IL-4 and IL-13 in susceptibility following *L. major* infection (13). Collectively, these results indicated that along with IL-4, IL-13, and other factors are involved in the control of Th2 cell differentiation and *L. major* susceptibility (14).

In addition, several lines of evidence suggest that IL-4 may be needed for Th1 cell differentiation. Unlike what was observed following *L. major* infection, IL-4-deficient mice failed to develop Th1 cells in response to infection with *Candida albicans* (15) suggesting a potential role for endogenous IL-4 in Th1 cell differentiation and protective antifungal response. Furthermore, local injection of exogenous recombinant IL-4 within the first 8 h of *L. major* infection in BALB/c mice was sufficient to modify the development of the immune response from an otherwise Th2 immune response into a protective type-1 Th1 response (16). It was hypothesized that IL-4, by acting on dendritic cells, induced their IL-12 secretion (16), a process that had previously been reported on macrophages and DCs *in vitro* (17–19). In addition, dendritic cell-specific IL-4Rα-deficient mice on the BALB/c genetic background developed larger lesions and increased Th2 response, suggesting some protective role for endogenous IL-4 acting on DCs during *L. major* LV39 and IL-81 infection (20). Collectively, these studies suggested that within the first hours of *L. major* infection the transient presence of IL-4 could contribute to the differentiation of CD4^+^ Th1 cells. In this line, skin keratinocytes present in the footpad of mice infected with *L. major* subcutaneously were identified as an early IL-4 source contributing to the launching of CD4^+^ Th1 cell differentiation (21). Interestingly, in that study, IL-4 transcription appeared restricted to keratinocytes from C57BL/6 mice and only low IL-4 mRNA levels were observed in BALB/c keratinocytes. Moreover, in the same study, the upregulation of IL-4 mRNA observed in C57BL/6 keratinocytes was shown to be restricted to a very small time window at the onset of infection. Finally, impaired Th1 cell development was observed in C57BL/6 mice following blocking of IL-4 protein with an anti-IL-4 mAb at the cutaneous infection site (21). Targeting IL-4 at the infection site could be of potential interest in the design of vaccines.

Here, we investigated the role of skin IL-4Rα signaling, more specifically the contribution of keratinocyte-derived IL-4Rα signaling during the first days of *L. major* infection and its subsequent impact on the development of a protective type-1 immune response in C57BL/6 mice. To this end, we generated C57BL/6 mice specifically deficient in IL-4Rα in their keratinocytes (KRT14^Cre^IL-4Rα^−/lox^). As IL-4 and IL-13 share a common signaling pathway through the IL-4Rα the combined role of both cytokines could be studied in these mice. Following infection with *L. major* in the ear dermis or subcutaneously in the footpad, these mice were fully able to control their lesion size and develop Th1 cells. Furthermore, using IL-4 reporter mice, we identified mast cells as the only skin cells transcribing but not producing IL-4 in C57BL/6 skin early in infection. Our data demonstrate that the presence of IL-4 and IL-4 signaling in the skin during the first days after *L. major* infection are not required for the development of a protective Th1 immune response in C57BL/6 mice.

## Materials and Methods

### Mice

C57BL/6 mice were purchased from Charles Rivers. *4get*, and KN2 mice (22, 23) were a gift from Markus Mohrs, Trudeau Institute, USA. Keratinocyte cell-specific 4get/KN2 mice were obtained by the breeding of 4get and KN2 mice. IL-4Rα-deficient (KRT14^cre^IL-4Rα^−/lox^) mice on the C57BL/6 genetic background were generated using the *Cre/lox*P system. Briefly, KRT14^cre^ mice on the BALB/c genetic background (Jackson Laboratory) were first backcrossed on the C57BL/6 genetic background for nine generations. The progeny were crossed with IL-4Rα^−/−^ C57BL/6 mice and transgenic IL-4Rα^lox/lox^ mice (24) to generate hemizygous KRT14^cre^IL-4Rα^−/lox^ C57BL/6 mice. Genotyping of KRT14^cre^IL-4Rα^−/lox^ C57BL/6 mice was performed using specific primers: KRT14 P1, 5’- TTC CTC AGG AGT GTC TTC GC; KRT14 P2, 5’- GTC CAT GTC CTT CCT GAA GC; KRT14 P3, 5’- CAA ATG TTG CTT GTC TGG TG; KRT14 P4, 5’- GTC AGT CGA GTG CAC AGT TT. Characterization was also performed by flow cytometry using the anti-IL-4Rα mAb [anti-CD124, anti-IL-4Rα-PE from (BD, Pharmingen)]. Mice were bred under pathogen-free conditions at the Epalinges Center. 5- to 8-week-old females were used.

### Ethics Approval

All animal experimental protocols were approved by the veterinary office regulations of the State of Vaud, Switzerland, authorization 1266.6-7 to FTC and performed in compliance with Swiss ethics laws for animal protection. All mouse experiments performed at the University of Cape Town were performed in strict accordance with the South African national guidelines, as well as the Animal Research Ethics Committee of the Faculty of Health Sciences, University of Cape Town (license no. 015/034). All efforts were made to minimize and reduce suffering of animals.

### Parasites and Infections

*Leishmania major* (LV39, MRHO/Sv/59/P strain) or in selected experiments GFP-expressing *L. major* IL-81 (MHOM/IL/81/FEBNI) parasites were cultured at 26°C in complete M199 medium (10% fetal bovine serum, 4% HEPES, 1% penicillin, streptomycin, and neomycin). 10^4^ or 2 × 10^5^ *L. major* metacyclic promastigotes were prepared from confluent stationary phase promastigotes by Ficoll gradient density centrifugation as previously described (25) and injected in the ear dermis or footpad. Lesion development was monitored using Vernier caliper, ear lesion diameter was measured for intradermal infection. For IL-4 depletion, 2 × 10^5^ metacyclic parasites (±1 µg 11B11 antibody) were needle injected into the ear dermis in iDMEM in a final volume of 10 µL, or in the hind footpad in a volume of 50 µL. Four hours later, 1 µg of 11B11 mAb was injected intradermally (i.d.) in a final volume of 10 µL. For the induction of a Th2 response in C57BL/6 4get/KN2 mice, 1 mg of αIFNγ (XMG1.2) and αIL-12 were injected intraperitoneally 24, 2 h prior and 48 h post intradermal infection of 2 × 10^5^ *L. major* metacyclic promastigotes.

### Determination of Antibody Response

*Leishmania major*-specific IgG1 and IgG2c levels were determined by ELISA in the sera of mice harvested at the termination of the experiment, as previously described (26), Biotinylated goat anti-mouse IgG2c (Southern Biotech) and biotinylated rat anti-mouse IgG1 (BD Pharmingen) were used. Plates were read at the optical density of 490 nm. Titration curve were performed for all samples.

### Isolation of dLN, Spleen, and Ear Mouse Cells

Infected ears, spleen, and dLNs were isolated and processed to single cell suspensions. Briefly, ears were recovered, homogenized in iDMEM containing 0.2 mg/mL Liberase TL (Roche) for 2 h at 37°C and then homogenized and filtered using 40-µm filters (Falcon). dLN and spleen cells were isolated, homogenized, and stained for flow cytometry analysis. For intracellular cytokine staining, 1 × 10^6^ dLN cells were stimulated with 50 µg/mL PMA, 500 µg/mL Ionomycin, and 1 µg/mL Brefeldin A for 4 h prior staining. Live parasites were determined by limiting dilution assay (LDA) as described previously (27).

### Cytokine Production

10^6^ dLN cells were cultured in presence or absence of UV-irradiated stationary phase parasites (parasites were exposed to UV.C for 3 min, at a distance of 10 cm from the UV lamp) *L. major* promastigotes (MOI of 5:1) for 72 h at 37°C. Supernatants were recovered and IL-4 production measured by ELISA following manufacturer’s instructions (BD Biosciences; R&D). IFNγ secretion was detected using a homemade ELISA kit as previously described (28).

### Derivation of Mouse Primary Keratinocytes

Primary keratinocytes were isolated from the skin of adult mice tail. Briefly, the skin from the tail was collected and incubated at 4°C for 16 h in a solution of 5 U/mL Dispase (STEMCELL) supplemented with 1% PNS (Gibco) and 0.5% Gentamicin (Gibco). The epidermis was separated from the dermis and treated with 0.2% trypsin (Gibco) for 5 min at 37°C. The reaction was stopped with FCS, and cells were collected by crushing the epidermis with a 100-µm cell strainer (Falcon). 7.5 × 10^5^ cells/mL were plated in a 6-well plate coated with Type I collagen (STEMCELL). Cells were grown for 8 days at 37°C in CnT-57.S medium (CELLnTEC) supplemented with 1% PNS and 0.5% Gentamicin. Medium were removed and cells detached with 200 µL 0.2% trypsin for 5 min at 37°C. Cells were counted and plated at 0.3 × 10^6^ cells/mL in a 96-well plate coated with Type I collagen.

### Flow Cytometry

Stained cells were analyzed using a BD LSR II-SORP or Fortessa system (Becton Dickinson) and analyzed with FlowJo software (Tree Star). The following anti-mouse mAbs were used: CD45- PerCPCy5, CD45-APC, CD8-APC, anti-IFNγ-PECy7, and anti-IL-4-FITC (BD Biosciences), CD4-AF700, CD11b APC-eFluor780, and c-kit PECy7 all from (eBiosciences). Anti human CD2 (BioLegend) was used to detect IL-4 production in KN2 mice. To detect IL-4Rα on primary keratinocytes, the following anti-mouse mAbs were used: CD124, anti IL-4Rα-PE (BD, Pharmingen), and CD49f-Brillant Violet 421 (BioLegend). Imaging flow cytometry was performed using an ImageStream cytometer (Amnis, Millipore) at low speed. Data were analyzed with the IDEAS software.

### Statistics

All *p* values were determined with Prism software (GraphPad Software, Inc.) using the Student’s *t*-test for unpaired data or paired data, following prior testing for normal distribution. The degree of significance was indicated as *p < 0.05, **p < 0.01, and ***p < 0.001.

## Results

### Absence of IL-4Rα Signaling in Keratinocytes Does Not Prevent the Development of a Protective Type-1 Immune Response

**Figure 1:**
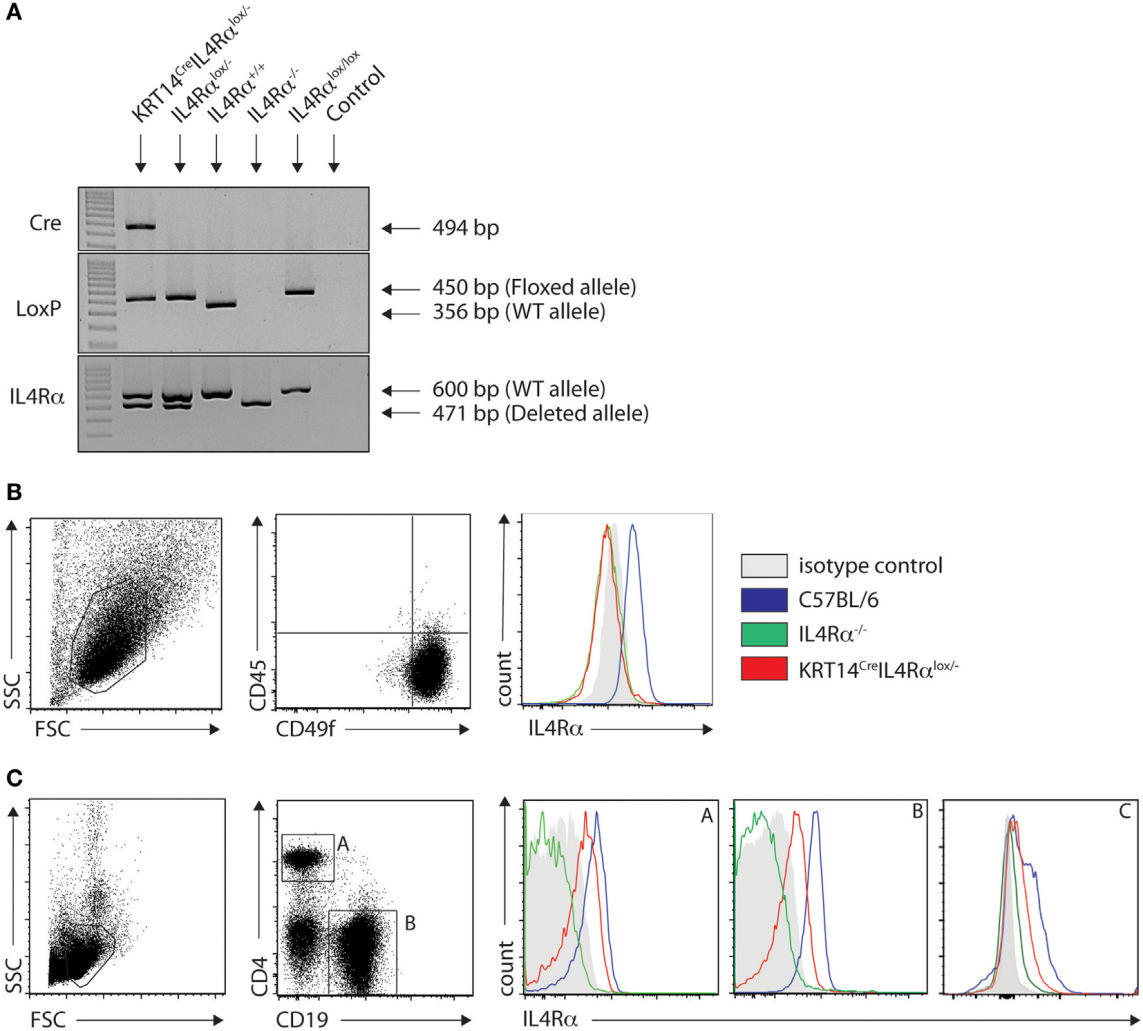
Characterization of KRT14^cre^IL-4Rα^−/lox^ C57BL/6 mice. **(A)** Genotyping of KRT14^cre^IL-4Rα^−/lox^ mice, IL-4Rα^−/lox^, IL-4Rα^+/+^, IL-4Rα^lox/lox^, and negative control (H_2_O) is shown. The yielded PCR products in base pairs are indicated in the figure. **(B)** IL-4Rα cell surface expression was analyzed by flow cytometry on primary keratinocytes of the indicated mice. **(C)** IL-4Rα expression was analyzed in splenic CD4^+^ T cells (A), CD19^+^ B cells (B) and F480^+^ (C) macrophages of the indicated mice. Data shown are representative of three independent experiments.

To investigate the impact of IL-4Rα signaling on keratinocytes in a protective Th1 cell differentiation against cutaneous leishmaniasis, we generated mice on the C57BL/6 genetic background that are genetically deficient in IL-4Rα expression on keratinocytes (KRT14^Cre^IL-4Rα^−/lox^) using the Cre/LoxP recombination system under control of the *KRT14* locus. In these mice, keratinocytes cannot respond to IL-4 and IL-13, while other cells can. The absence of skin IL-4Rα expression was verified by PCR of naïve ears. KRT14^Cre^IL-4Rα^−/lox^ expressed both the deleted and WT IL-4Rα allele in addition to the LoxP and Cre bands. The genotype was also validated for IL-4Rα^−/lox^, IL-4Rα^+/+^ (C57BL/6 wild type) control mice as well as for IL-4^−/−^ on the C57BL/6 genetic background (Figure 1A). The selective absence of IL-4Rα on KRT14^Cre^IL-4Rα^−/lox^ keratinocytes was further analyzed by flow cytometry on primary keratinocytes derived from the skin of these mice. IL-4Rα expression was not detected on CD45^−^CD49f^+^ keratinocytes of KRT14^Cre^IL-4Rα^−/lox^ mice. As controls, expression of IL-4Rα was detected on keratinocytes of wild-type C57BL/6 mice and was absent on keratinocytes of IL-4Rα^−/−^ mice (Figure 1B). As expected, expression of IL-4Rα was, however, detected in splenic CD4^+^ T cells, CD19^+^ B cells, and F480^+^ macrophages of KRT14^Cre^IL-4Rα^−/lox^ mice (Figure 1C). The slightly lower IL-4Rα surface expression levels observed in KRT14^Cre^IL-4Rα^−/lox^ CD4^+^ T cells, CD19^+^ B cells and F480^+^ macrophages compared to those observed in C57BL/6 wild-type cells are in line with the IL-4Rα^−/lox^ genotype of the control mice. No IL-4Rα expression was observed in IL-4Rα^−/−^ CD4^+^ T cells and CD19^+^ B cells. These data show that KRT14^Cre^IL-4Rα^−/lox^ mice do not express IL-4Rα selectively on keratinocytes.

**Figure 2:**
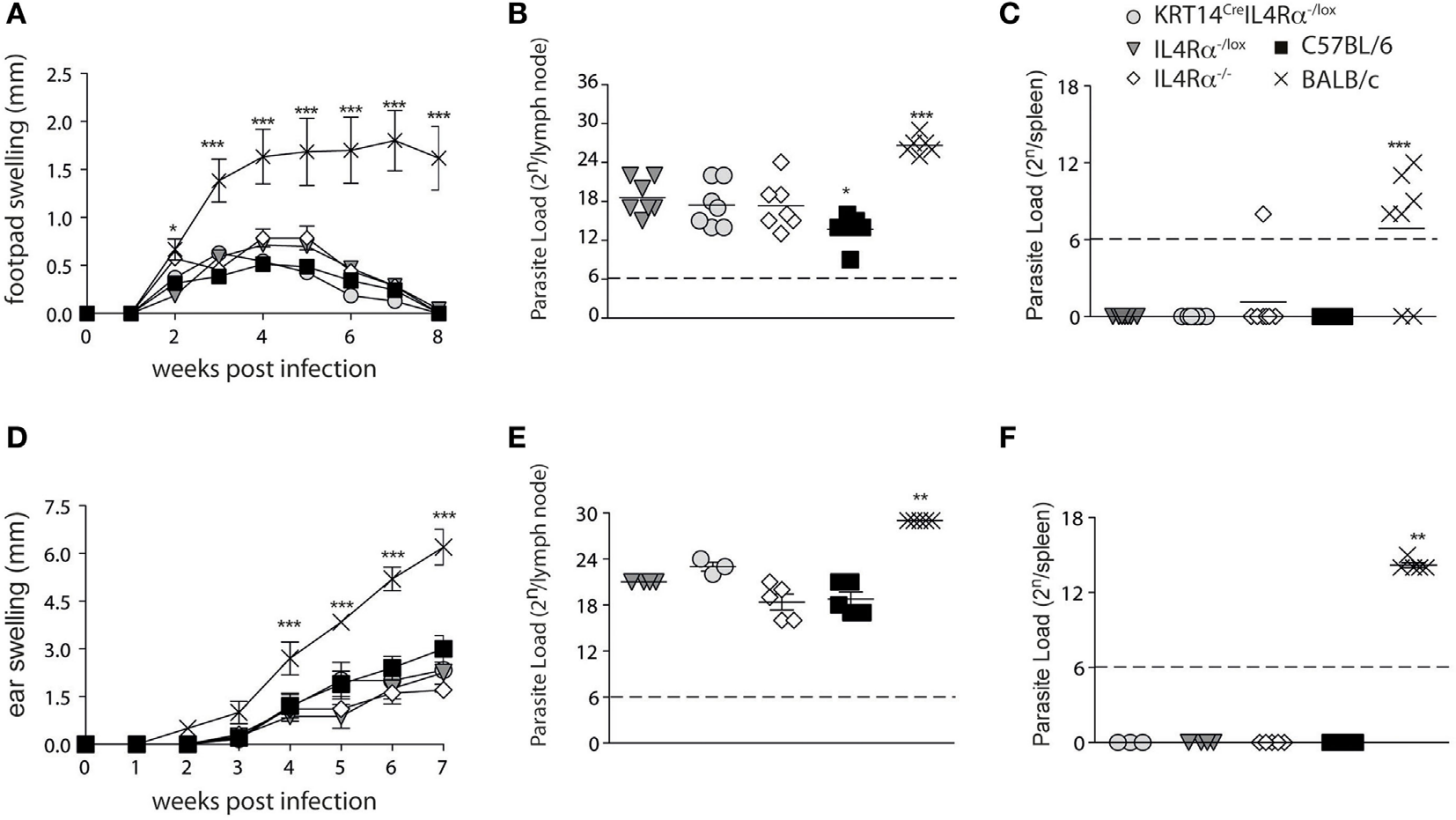
Mice on a C57BL/6 genetic background that are deficient for IL-4Rα in their keratinocytes or on all cells are fully able to control their lesion size and parasite load following s.c. or i.d. *Leishmania major* infection. **(A)** KRT14^cre^IL-4Rα^−/lox^, IL-4Rα^−/lox^, C57BL/6, and IL-4Rα^−/−^ mice were infected s.c. with 2 × 10^5^ metacyclic *L. major* LV39 and lesion size was monitored. **(B)** Parasite load in draining lymph nodes (dLNs) and **(C)** spleen was analyzed by limiting dilution assays (LDAs) 8 weeks postinfection. **(D)** The indicated mice were infected in the ear dermis with 10^4^ *L. major* and lesion development monitored. **(E)** Parasite load in the dLN or **(F)** spleen was analyzed by LDAs 7 weeks postinfection **p < 0.01 ***p < 0.001 compared to BALB/c. *p < 0,05 C57BL/6 compared to IL-4Rα^−/lox^, IL-4Rα^−/−^, and KRT14^cre^IL-4Rα^−/lox^. These data are representative of two independent experiments.

To investigate if IL-4Rα signaling in keratinocytes could have an impact on the local microenvironment driving Th1 differentiation during *L. major* infection, KRT14^Cre^IL-4Rα^−/lox^ on the C57BL/6 genetic background were infected subcutaneously in the footpad with 2 × 10^5^ *L. major*. Lesion development and parasite load in the dLNs and spleen were analyzed and compared to lesion developing in control IL-4Rα^−/lox^, IL-4Rα^+/+^, and IL-4Rα^−/−^ mice all on the C57BL/6 genetic background. *L. major* susceptible BALB/c mice were similarly infected and used for comparison. No difference in lesion development was observed between all groups of mice on the C57BL/6 genetic background, and BALB/c mice developed a significantly larger lesion (Figure 2A). Parasite load was also similar in the dLNs of these mice and no parasite dissemination to the spleen was observed, while significantly higher dLN parasite load and the presence of parasite dissemination to the spleen were observed in BALB/c mice (Figures 2B,C). Phlebotomine sand flies deposit significantly lower dose of parasites in the mammalian dermis, and it was previously reported that the cellular recruitment differ between subcutaneous infection in the footpad or intradermal infection in mouse ear dermis (29). To assess a potential role for IL-4Rα signaling in keratinocytes on disease outcome in more physiological conditions, we needle inoculated mice with 1 × 10^4^ *L. major* LV39 metacyclic parasites, in the ear dermis of KRT14^Cre^IL-4Rα^−/lox^ mice. Lesion development and parasite load were compared to those observed in similarly infected IL-4Rα^−/lox^, IL-4Rα^−/−^ mice, IL-4Rα^+/+^ mice all on the C57BL/6 genetic background. No difference in lesion size was observed in all groups of mice. In contrast, similarly infected BALB/c mice developed non-healing lesions (Figure 2D). All groups of mice on the C57BL/6 genetic background also controlled parasite load as assessed in the dLN and no parasite dissemination to the spleen was observed while similarly infected BALB/c mice showed higher parasite load in dLN and parasite disseminated to the spleen (Figures 2E,F). These data demonstrate that IL-4Rα signaling in keratinocytes is not required for control of lesion development and parasite burden. In addition, total lack of IL-4Rα signaling did not have an impact on the resolution of the infection. The control of parasite load in KRT14^Cre^IL-4Rα^−/lox^ and IL-4Rα^−/−^ mice suggested that an efficient protective Th1 immune response could develop in these mice in absence of IL-4Rα signaling.

### Mice Deficient in IL-4Rα Signaling in Keratinocytes Are Capable to Mount a Th1 Cell Response following *L. major* Infection

**Figure 3:**
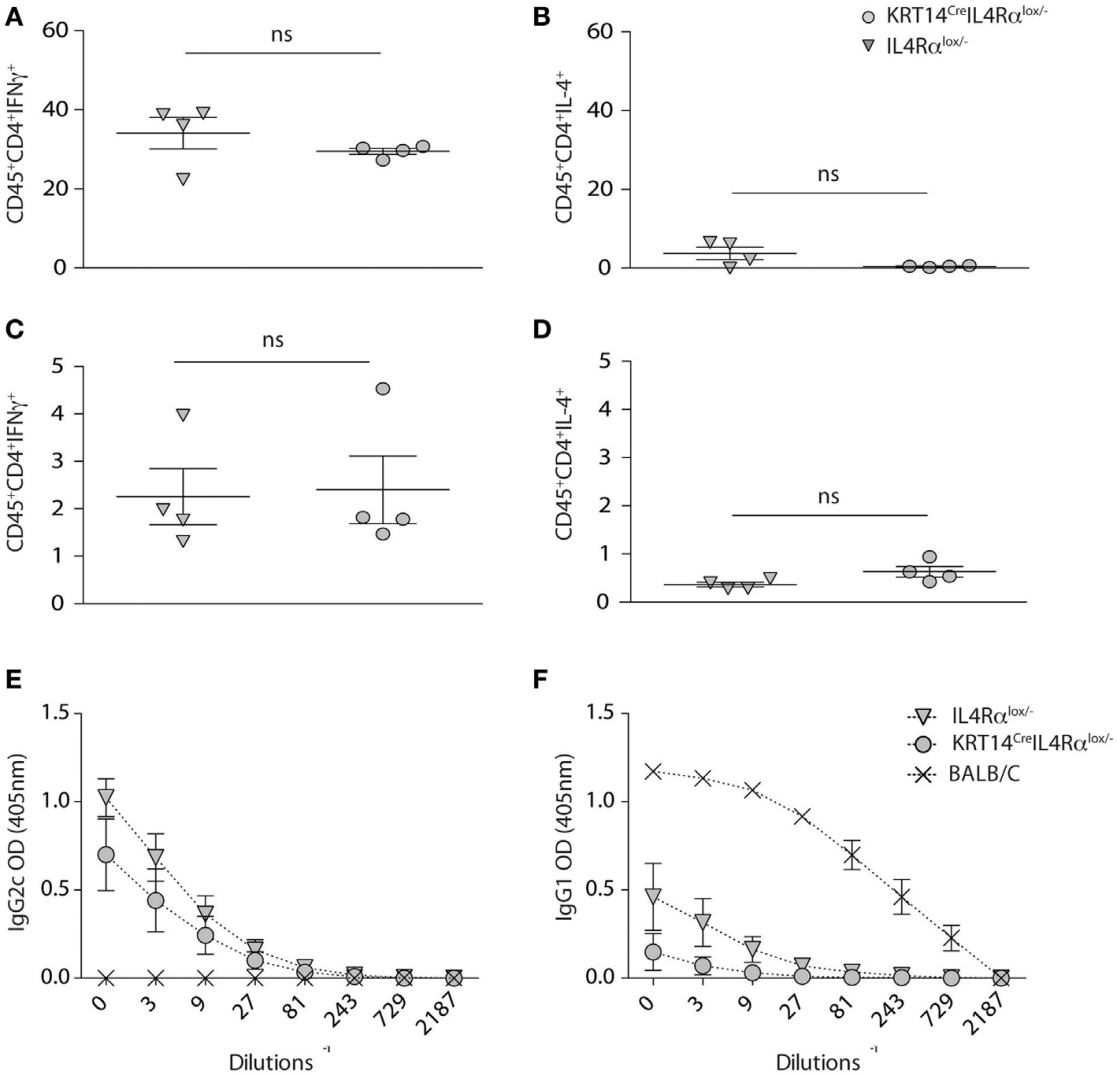
Absence of IL-4Rα signaling selectively on keratinocytes does not impair T helper (Th) 1 cell differentiation. **(A,B)** KRT14^cre^IL-4Rα^−/lox^, IL-4Rα^−/lox^ were infected i.d. in the ear pinna with 10^4^ *Leishmania major* parasites in the ear dermis. Seven weeks postinfection, the frequency of CD4^+^IFNγ^+^ and CD4^+^IL-4^+^ T cells at the site of infection (ears) was analyzed by flow cytometry. **(C,D)** Similar analysis was performed on draining lymph node cells at the same time point p.i. **(E)** Serum from these mice was analyzed and compared to that of BALB/c mice similarly infected for the presence of *L. major*-specific IgG2c and **(F)** IgG1. Data shown are representative of two independent experiments.

To further investigate in details if IL-4Rα signaling in keratinocytes impacted Th1 cell differentiation during cutaneous leishmaniasis, KRT14^Cre^IL-4Rα^−/lox^ mice and control IL-4Rα^−/lox^ were infected with 10^4^ *L. major* (LV39). Four weeks after infection, the frequency of IFNγ and IL-4 producing cells was analyzed by flow cytometry at the site of infection. A high frequency of CD4^+^IFNγ^+^ was detected in the ear dermis of KRT14^Cre^IL-4Rα^−/lox^ mice, with no difference compared to that measured in similarly infected IL-4Rα^−/lox^ control mice (Figure 3A). Only very low levels of CD45^+^CD4^+^IL-4^+^ cells were detected in both groups (Figure 3B). The frequency of CD4^+^IFNγ^+^ cells observed in dLN cells of both groups of mice was also similar (Figure 3C) and more elevated than the frequency of CD45^+^CD4^+^IL-4^+^ cells observed in dLN cells (Figure 3D) revealing that the CD4^+^ Th1 cells are able to differentiate in absence of IL-4Rα signaling in keratinocytes.

In line with the elevated levels of IFNγ detected at the site of infection and in dLN cells, high levels of serum IgG2c were observed in KRT14^Cre^IL-4Rα^−/lox^ and control IL-4Rα^−/lox^ mice (Figure 3E). BALB/c mice showed elevated levels of IgG1, correlating with the high production of IL-4 observed in their CD4^+^ T cells, while sera from KRT14^Cre^IL-4Rα^−/lox^ and control mice did not show significant IgG1 levels (Figure 3F), in line with the absence of Th2 cells detected in dLN and at the site of infection. Similar data were obtained following s.c. infection in the footpads with a higher dose of parasites (2 × 10^5^ *L. major*, data not shown). Taken together, these results show that the absence of IL-4Rα signaling in keratinocytes does not impair the development of a protective Th1 type of immune response during early *L. major* infection.

### Detection of IL-4 Expressing Cells in the Ear Dermis of C57BL/6 Mice 1 Day after *L. major* Infection

**Figure 4:**
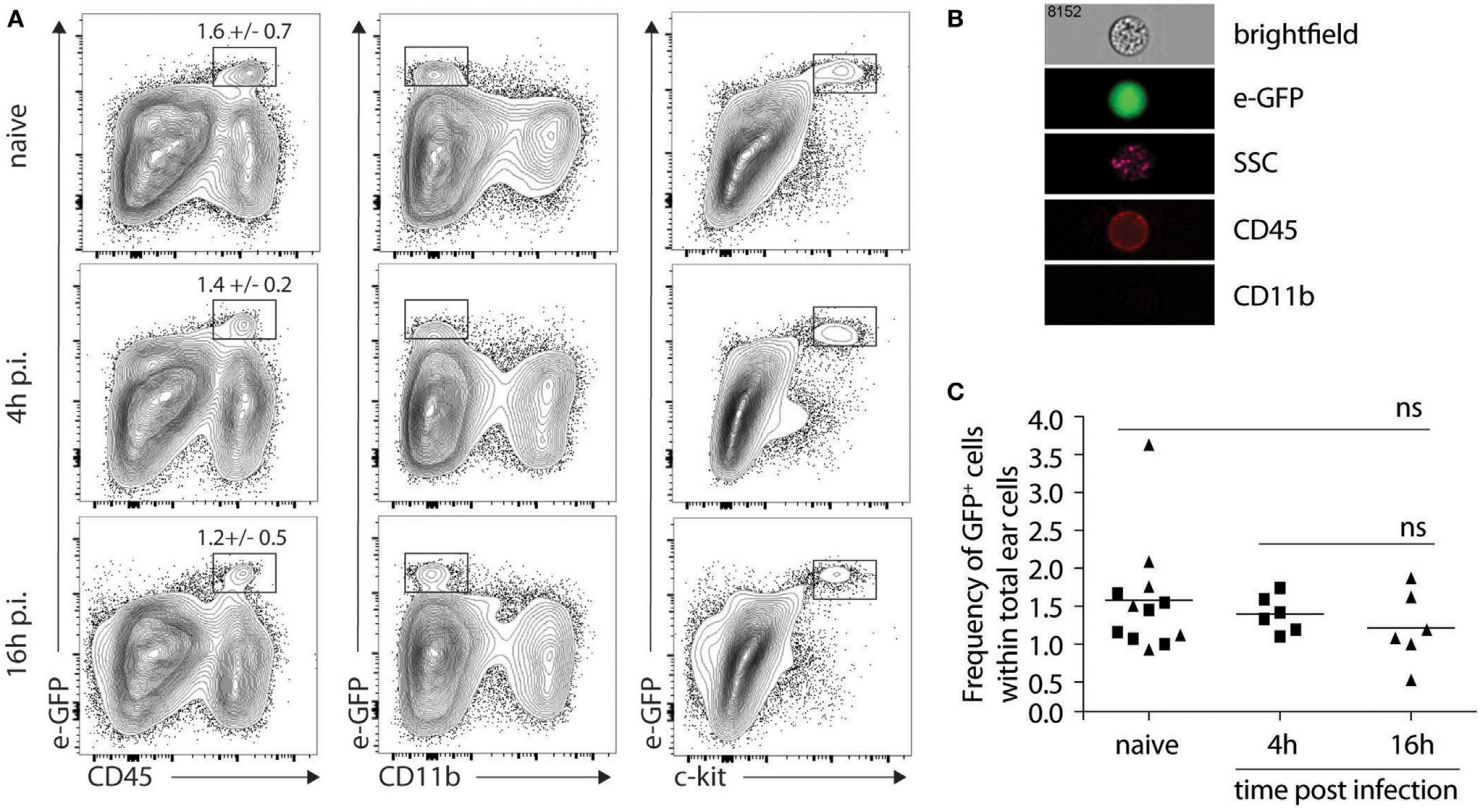
Mast cells express *il-4* in naive and *Leishmania major*-infected ears of C57BL/6 mice. **(A)** C57BL/6 4get mice were inoculated i.d. into the right ear with 2 × 10^5^ metacyclic *L. major*. Ears were recovered 4 or 16 h p.i. processed and analyzed by flow cytometry for e-GFP, CD45, CD11b and c-kit surface expression. As negative controls, naïve ears were also isolated and similarly processed **(B)**. Four hours postinfection, ears were similarly processed, stained with CD45 and CD11b, and analyzed by imaging flow cytometry. The e-GFP positive cells were all CD45^+^CD11b^−^, and showed a high granulosity (SSC) and a bright field phenotype typical of mast cells. A representative picture is shown. n.s: non-significant. **(C)** Frequency of e-GFP positive cells among total ear cells for 4get C57BL/6 at 0 (naïve), 4, or 16 h (▲) p.i. The data are pooled from three independent experiments.

The lack of impact of IL-4Rα signaling on lesion development, parasite control, and the differentiation of CD4^+^ Th1 cells at the site of infection and in the dLN firmly demonstrated that IL-4 signaling on keratinocytes was not involved in Th1 cell differentiation following *L. major* infection. However, it did not rule out an early role for keratinocyte-derived IL-4 in Th1 cell differentiation. To visualize the presence of *il-4* mRNA expressing cells in the mouse ear dermis cells of *L. major*-infected mice, we used the bicistronic IL-4 reporter (4get) mice on the C57BL/6 genetic background (22). We first determined if *il-4* mRNA expression was detectable in naive ear skin of C57BL/6 mice. A clear e-GFP-positive population was observed selectively in the CD45^+^ hematopoietic population. These cells did not express CD11b at their cell surface and stained positive for c-kit, a marker specific for mast cells (Figure 4A). Further analysis by imaging flow cytometry confirmed that the e-GFP *il-4*-expressing cells corresponded to mast cells characterized by the expression of CD45, the lack of expression of CD11b, their large size (average of 14.5 µm of diameter) and the presence of numerous granules (Figure 4B). We then analyzed the modulation of *il-4* mRNA expressing cells 4 and 16 h following i.d. infection with *L. major*, at a time when expression of *il-4* mRNA by keratinocytes was reported to be the highest following subcutaneous infection in the footpad (21). No e-GFP^+^ cells were detectable in the CD45-negative cell population, suggesting that keratinocytes did not express detectable levels of *il-4* mRNA at the time analyzed. Mast cells were the only cell population where *il-4* mRNA, as assessed by e-GFP expression, was detectable 4 and 16 h p.i. (Figure 4A lower panels). The frequency of e-GFP^+^ cell (approximately 1.5% of total ear cells) between infected and the contralateral uninfected ear was analyzed for each mouse. It did not vary between infected and uninfected ears at the analyzed time points (Figure 4C). These data show that most of the *il-4* mRNA expressing cells in the ear during the first 16 h post infection are mast cells and that *L. major* does not modulate *il-4* mRNA expression in these cells early during infection.

### Mast Cells Do Not Produce IL-4 upon *L. major* Infection

**Figure 5:**
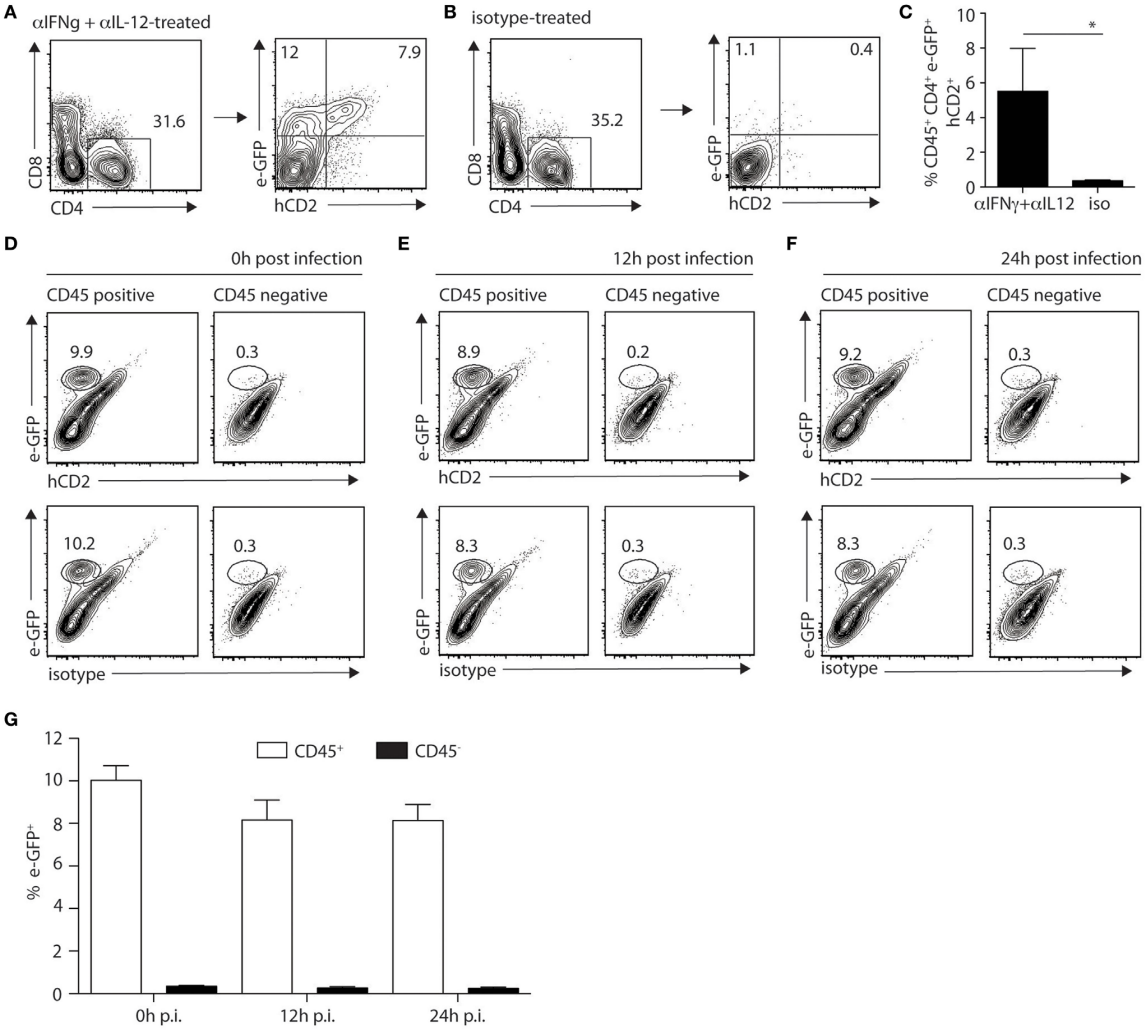
IL-4 production is not detectable locally during the first hours of *Leishmania* infection in mice on the C57BL/6 genetic background. **(A)** C57BL/6 KN2/4get mice were injected intraperitoneally with anti-IFNγ and anti-IL-12 24 and 2 h before and 48 h after infection with 2 × 10^5^ *Leishmania major* i.d. in the ear. Four weeks p.i. ears were isolated and digested prior to labeling with anti-CD4 and anti-CD8 mAbs. CD4^+^ T cells were analyzed for the expression of *il-4* detected by e-GFP and protein production of IL-4 detected by surface staining with a huCD2 mAb. **(B)** C57BL/6 KN2/4get mice were similarly infected but injected with an isotype control mAb at the onset of infection. Four weeks postinfection, IL-4 expression and production were analyzed by detection of e-GFP and huCD2 staining, respectively. **(C)** Bar graph gives the frequency of CD45^+^CD4^+^eGFP^+^hCD2^+^ cells (means ± SD of 3–5 animals per group) *p < 0.05. These are representative of three experiments. **(D–F)** C57BL/6 KN2/4get mice were inoculated into the right ear with 2 × 10^5^ metacyclic *L. major*. Ears were recovered 0, 12, and 24 h p.i., processed, and stained with CD45, human CD2, or its isotype control mAbs and analyzed by flow cytometry to detect the expression (e-GFP) and production (huCD2^+^) of IL-4. Representative flow cytometry profiles are shown **(D–F)** and **(G)** the bar graph shows the frequency of e-GFP^+^CD45^+^ and e-GFP^+^CD45^−^ cells (mean ± SD of 4 animals per group). Data are representative of three experiments.

Mast cells appeared to be the major dermal cells expressing IL-4 early after infection, suggesting that this source of IL-4 could play a role locally. To visualize IL-4 cytokine production, C57BL/6 KN2 (knockin huCD2) IL-4 reporter mice that specifically report the production of IL-4 protein were used. Cells producing IL-4 express a fragment of the human CD2 molecule at the cell surface making it easily detectable by flow cytometry (23). We crossed these KN2 mice with 4get mice to simultaneously detect IL-4 expression and production. First, to ensure that digestion of the ear would not impact IL-4 detection, KN2/4get mice, all on the C57BL/6 genetic background, were treated with anti-IFNγ and anti-IL-12 mAbs at the onset of infection to induce Th2 cell differentiation. Four weeks post infection, ears from mAb-depleted or control mice were isolated, processed, and analyzed by flow cytometry. Treatment with anti-IFNγ and anti-IL-12 promoted the differentiation of Th2 cells. *il-4* mRNA expression (e-GFP^+^ cells) and IL-4 protein production (detected by hCD2 expression) were easily detectable (7.9%) in the ear dermis of CD4^+^ T cells (Figures 5A,C). Minimal IL-4 expression or production (0.4%) was detectable in ear cells of similarly infected KN2/4get mice treated with a control mAb (Figure 5B) in line with the differentiation of Th1 cells in these mice. 4get/KN2 naïve ears (0 h p.i.) showed detectable *il-4* mRNA expression in CD45^+^ but not in CD45^−^ cells and no IL-4 proteins as no huCD2^+^ cells were detected in either population, suggesting a lack of IL-4 production (Figure 5D). Infection did not change the frequency of cells transcribing *il-4* and no IL-4 production was observed 12 and 24 h after *L. major* infection (Figures 5E–G). These data suggest that ear skin mast cells transcribe the *il-4* gene but do not produce detectable IL-4 proteins at steady-state conditions, and *L. major* does not induce IL-4 production in the ear dermis early during infection.

Taken together, these results demonstrate that during the first hours of infection, *il-4* gene expression can be detected at the infection site selectively in mast cell, but *il-4* mRNA expression was not modulated during *L. major* infection (analyzed at 0, 4,12,16, and 24 h p.i.). Furthermore, no IL-4 protein production was detectable in the ear dermis during the first 24 h after *L. major* infection.

### Local Neutralization of IL-4 Does Not Impair Th1 Development in Response to *L. major* Infection

**Figure 6:**
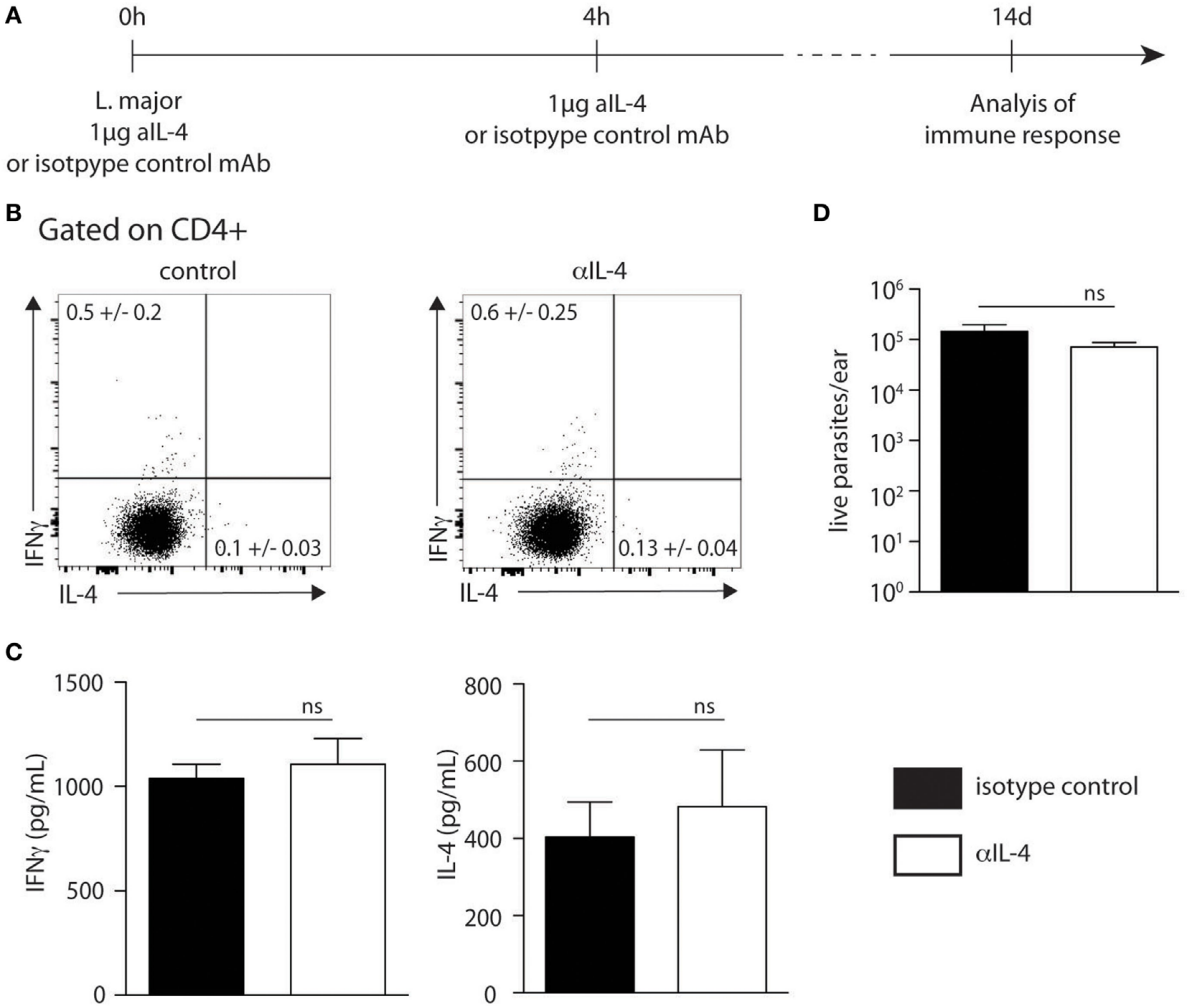
Neutralization of IL-4 prior to infection with *Leishmania major* i.d. does not impact T helper (Th) 1 immune response. **(A)** Neutralization of IL-4 strategy. The anti-IL-4 mAb (11B11) or an isotype control mAb was injected intradermally simultaneously with the infection of 2 × 10^5^ metacyclic *L. major* in the ear dermis. Four hours later, another injection of anti-IL-4 was given i.d. T helper immune response was analyzed 14 days later. **(B)** Two weeks postinfection, cervical draining lymph node (dLN) cells were recovered. The levels of IFNγ and IL-4 were assessed by intracellular staining and analyzed by flow cytometry. A representative plot is shown. **(C)** The levels of IFNγ and IL-4 secretion was quantified by ELISA in supernatants of dLN cells restimulated with UV-irradiated *L. major*. Mean cytokine expression ± SEM is given (*n* ≥ 3 mice per group). The data are pooled from two independent experiments. **(D)** Parasite load in the ear analyzed by limiting dilution assay 2 weeks p.i. Data presented as mean ± SEM (*n* ≥ 3 mice per group). Data shown are representative of two independent experiments. n.s: non-significant.

To further investigate if skin-derived IL-4 played a role in Th1 cell differentiation, C57BL/6 wild-type mice were infected with 2 × 10^5^ *L. major* in the ear dermis following the protocol described in Figure 6A, which is similar to that described previously (21). Two weeks post infection, the frequency of CD4^+^IFNγ^+^ and CD4^+^IL-4^+^ dLN T cells represented in average 0.6 and 0.13% of total CD4^+^ T cells, respectively. These values were similar to those obtained in mice similarly infected but treated with a control mAb (Figure 6B). The levels of IFNγ and IL-4 secretion in dLN cells stimulated *ex vivo* with UV-treated *L. major* 2 weeks after infection also showed no significant differences between the anti-IL-4 and control groups (Figure 6C). In line with these data, no difference in parasite load was observed between groups depleted or not of IL-4 at the onset of infection (Figure 6D). These results show that neutralization of IL-4 locally in the ear dermis during the first hours of infection at the site of infection does not have an impact on the development of the Type-1 immune response during cutaneous leishmaniasis.

## Discussion

The study of the early events leading to the differentiation of Th1 or Th2 cells is of importance and has relevance in the design of vaccines generated against pathogens inducing a specific Th type of immune response. IL-4 and its signaling through interaction with the IL-4/IL-13 receptor has been described as an important cytokine favoring the differentiation of CD4^+^ Th2 cells and type 2 immunity (30). However, several lines of evidence suggest that IL-4 may also be involved in Th1 cell differentiation (15–18, 31, 32). In this line, it was reported that following s.c. injection in the hind footpad of a very high dose (2 × 10^7^) of *L. major* parasites, keratinocytes transiently upregulated *il4* mRNA mostly within the first 16 h of infection. Blocking of IL-4 at the infection site impaired IFNγ secretion by dLN T cells 7 days later and increased IL-4 secretion by dLN cells 1 week post infection (21).

In an attempt to analyze if early after *L. major* inoculation, IL-4 secretion by keratinocytes could act in an autocrine way on these cells locally during the first days of infection, we generated mice genetically deficient in the IL-4Rα on keratinocytes. IL-4Rα hemizygosity (-/lox) was used to increase the probability of the floxed allele deletion by the Cre recombinase (24). IL-4 was previously shown to act on structural components of keratinocytes *in vitro*, a process that could be linked to epidermal structural problems (33). We show here that naive KRT14^Cre^IL-4Rα^−/lox^ mice are viable and have a normal phenotype. Upon infection with *L. major* i.d. in the ear pinna with a low or with a higher dose of parasites, mice with impaired IL-4Rα signaling on keratinocytes were able to resolve their cutaneous lesion, develop protective Th1 cells, and control parasite load. Similar data were obtained following infection of a higher dose of *L. major* subcutaneously in the footpad. These data demonstrate that following *L. major* infection IL-4Rα signaling in keratinocyte does not impair wound healing and is not required for the development of a protective type-1 immune response.

To directly visualize if IL-4 expression was induced in keratinocytes following infection with *L. major*, we used the well characterized IL-4-GFP reporter 4get mice (22). We detected *il-4*-transcribing cells in the ear dermis of naïve C57BL/6 mice and the majority of the positive cells were mast cells and not keratinocytes. Mast cells are tissue-resident cells and it was previously reported that mast cells from BALB/c mice constitutively express *il4* mRNA at steady state (34). The detection of e-GFP^+^ (*il-4* transcribing) mast cells in naïve ears detected here is thus in line with this study. Mast cell stimulation is required for these cells to secrete IL-4 protein. To verify if stimulated mast cells could be the source of the early IL-4 putatively present in C57BL/6 skin of *L. major*-infected mice, we used the KN2 reporter mice on the C57BL/6 genetic background, which allow the visualization of cells that produce IL-4 protein (23). Mast cells did not produce detectable IL-4 levels upon *L. major* infection within the first 24 h p.i. in KN2 mice. It was previously reported that C57BL/6 mice deficient in mast cells (C57BL/6 Cpa^Cre^) were perfectly able to mount a Th1 type of cell immune response following *L. major* infection (35). Together with our study, these results rule out a role for early mast cell-derived IL-4 in driving Th1 cell differentiation and resolution of lesion size and parasite load following *L. major* infection. In addition, *il4* transcription was not detectable in keratinocytes during the first day of *L. major* infection.

It cannot be excluded that a few cells may locally express *il-4* at low levels at steady state or upon *L. major* infection and that these cells were not detectable using *L. major*-infected 4get mice. Nevertheless, as no detectable IL-4-producing cells were observed in the skin of infected KN2 mice the possibility of IL-4 driving Th1 differentiation following infection with *L. major* is rather unlikely.

Several factors may explain the differences observed between the present report and previous studies reporting a role for IL-4 in Th1 differentiation following *L. major* infection. First, the parasite dose injected differed in the two studies. Ehrchen et al. reported *il-4* mRNA expression by keratinocytes during the first day after infection with a vey high dose (2 × 10^7^) of *L. major* inoculated s.c. in the hind footpad (21). During their blood meal, sand flies deposit a much lower number of parasites ranging from 10–10^4^ and it was shown that the size of the parasite inoculum can have an impact on the immune response (36). Here, following infection of reporter mice with 2 × 10^5^ *L. major*, no detectable levels of *il4* mRNA or IL-4 proteins were locally measured during the first days of infection. The parasite dose injected here is 100 times lower than that used in the study by Ehrchen et al., potentially explaining the differences observed. In addition, here, we used infective metacyclic *L. major* rather than stationary phase parasites to infect mice. Furthermore, the sensitivity of the microarray analysis used in the study by Ehrchen et al. is higher than that of the reporter system used here, as qPCR analysis can detect minute amounts of *il-4* mRNA. The absence of IL-4 proteins detected here suggests that if some IL-4 is produced locally following i.d. inoculation of parasites, it is in very low quantities.

A second major difference between the two studies is the site of parasite inoculation. Here, *L. major* was inoculated in the ear dermis, while in the Ehrchen’s study it was injected subcutaneously in the hind footpad. Recruitment of innate cells to the site of infection was recently shown to differ whether *L. major* was inoculated in the hind footpad or in the ear dermis of mice, with a distinct impact on the local microenvironment at the site of infection (29). However, here, we show that irrespective of the site of infection, the absence of IL-4Rα on keratinocytes in *L. major*-infected mice did not have any impact on lesion development and immune response.

Distinct *Leishmania* species elicit different immune response. In addition, distinct *L. major* strains also have diverse virulence [reviewed in Ref. (28)]. This could have explained the differences observed in the two studies. However, mice on the C57BL/6 genetic background with total abrogation of IL-4Rα on all cells were fully able to control lesion size and parasite load following either intradermal or subcutaneous infection with 2 × 10^5^ *L. major* of two different strains (LV39 or IR81), a process correlating with the development of functional Th1 cells in response to infection (here and *unpublished data*). Collectively, these data suggest that the difference observed in the two studies may not result from differences in the site of parasite inoculation or *L. major* strain virulence but rather come from differences in the parasite dose inoculated.

Distinct skin microbiota present in different animal facilities may also explain the differences observed between the previous and the present study as microbiota was shown to affect the immune response (37). This is, however, unlikely to be the explanation as the data presented here were obtained in two distinct laboratories with similar results. Furthermore, despite repeated attempts of blocking IL-4 locally using different parasite inoculation dose and injection scheme of anti-IL-4 mAb as well as distinct *L. major* parasite strains (here and *unpublished data*) we could not detect an impact of IL-4 blockade on the immune response and parasite control between 14 and 21 days post infection.

Another study reported the importance of early IL-4 in CD4^+^ Th1 differentiation (16). In this study, BALB/c mice infected with 2 × 10^5^ *L. major* LV39 stationary phase promastigotes were treated with recombinant IL-4 at the time and 8 h post *L. major* infection. Administration of a relatively high dose (1 µg) but not of a lower dose (0.1 µg) of IL-4 instructed resistance in these otherwise *L. major* susceptible BALB/c mice. One major difference with the present study is that BALB/c mice were used in that study. Furthermore, the dose of IL-4 injected locally may not correspond to cytokine levels present at the site of infection in *L. major*-infected C57BL/6 mice. If such levels of IL-4 protein had been present locally, it would have been detected here using the IL-4 reporter mice. Collectively, these and our data suggest that the early presence of IL-4 may have a different impact on the development of Th1 response depending on the dose of parasite inoculated, the genetic background of the mice, and the local amount of IL-4 protein present at the site of infection.

Altogether, our data provide strong evidence that IL-4 signaling in keratinocytes at the site of infection or in general, is not required for the differentiation of Th1 cells following *L. major* infection and that IL-4 protein production at the onset of infection is not required for Th1 cell differentiation in *L. major*-infected C57BL/6 mice.

## Ethics Statement

All animal experimental protocols were approved by the veterinary office regulations of the State of Vaud, Switzerland, authorization 1266.6-7 to FT-C and performed in compliance with Swiss ethics laws for animal protection. All mouse experiments performed at the University of Cape Town were performed in strict accordance with the South African national guidelines, as well as the Animal Research Ethics Committee of the Faculty of Health Sciences, University of Cape Town (license no. 015/034). All efforts were made to minimize and reduce suffering of animals.

## Author Contributions

MD, BH, MG, KP, BM-S, and RH performed the experiments and contributed to their analysis. BH, MG, and KP made the figures. FT-C, RG, and FB contributed to the conceptual design and provided funding for this project. FT-C wrote the manuscript with comments from BH, MD, KP, RH, RG, and FB.

## Conflict of Interest Statement

The authors declare that the research was conducted in the absence of any commercial or financial relationships that could be construed as a potential conflict of interest.
